# Loop-Mediated Isothermal Amplification Label-Based Gold Nanoparticles Lateral Flow Biosensor for Detection of *Enterococcus faecalis* and *Staphylococcus aureus*

**DOI:** 10.3389/fmicb.2017.00192

**Published:** 2017-02-10

**Authors:** Yi Wang, Hui Li, Yan Wang, Lu Zhang, Jianguo Xu, Changyun Ye

**Affiliations:** ^1^State Key Laboratory of Infectious Disease Prevention and Control, National Institute for Communicable Disease Control and Prevention, Collaborative Innovation Center for Diagnosis and Treatment of Infectious Diseases, Chinese Center for Disease Control and PreventionBeijing, China; ^2^Department of Microbiology, GuiZhou Medical UniversityGuiyang, China

**Keywords:** loop-mediated isothermal amplification, lateral flow biosensor, LAMP-LFB, *E. faecalis*, *S. aureus*, limit of detecton

## Abstract

The report describes a simple, rapid and sensitive assay for visual and multiplex detection of *Enterococcus faecalis* and *Staphylococcus aureus* based on multiple loop-mediated isothermal amplification (mLAMP) and lateral flow biosensor (LFB). Detection and differentiation of the *Ef0027* gene (*E. faecalis*-specific gene) and *nuc* gene (*S. aureus*-specific gene) were determined using fluorescein (FITC)-and digoxin-modified primers in the mLAMP process. In the presence of biotin- and FITC-/digoxin-modified primers, the mLAMP yielded numerous biotin- and FITC-/digoxin-attached duplex products, which were detected by LFB through biotin/streptavidin interaction (biotin on the duplex and streptavidin on the gold nanoparticle) and immunoreactions (FITC/digoxin on the duplex and anti-FITC/digoxin on the LFB test line). The accumulation of gold nanoparticles generated a characteristic red line, enabling visual and multiplex detection of target pathogens without instrumentation. The limit of detection (LoD), analytical specificity and feasibility of LAMP-LFB technique were successfully examined in pure culture and blood samples. The entire procedure, including specimen (blood samples) processing (30 min), isothermal reaction (40 min) and result reporting (within 2 min), could be completed within 75 min. Thus, this assay offers a simple, rapid, sensitive and specific test for multiplex detection of *E. faecalis* and *S. aureus* strains. Furthermore, the LAMP-LFB strategy is a universal technique, which can be extended to detect various target sequences by re-designing the specific LAMP primers.

## Introduction

Gram-positive bacteria, especially *Enterococcus faecalis* (*E. faecalis*) and *Staphylococcus aureus* (*S. aureus*), are among leading causes of nosocomial infection, such as bloodstream, surgical site and urinary tract infections (Filetoth, 2008). *E. faecalis*, which is natural inhabitant of the animal and human gastrointestinal tract, accounted for 80–90% of all enterococcal-associated infections (Peel et al., 2012). *S. aureus*, which is considered part of the normal microflora of microorganisms related with skin, throat and nose, is one of the most important pathogens in hospital- and community-acquired infections associated with high mortality (Foster et al., 2014). As such, reliable detection of *E. faecalis* and *S. aureus* is important for accurate diagnosis and instituting adequate antimicrobial therapy.

The conventional diagnostic assays, such as blood culture, were used as the gold standard for identifying *E. faecalis* and *S. aureus*, while it took more than 2 days to complete (Peters et al., 2004). Moreover, the analytical sensitivity of culture-based methods was markedly reduced if clinical samples were collected during antimicrobial therapy (Wallet et al., 2010). PCR and PCR-based detection of target pathogens directly in clinical samples has been established and was used as a fast alternative to the traditional culture techniques (Peters et al., 2007; Wang et al., 2016b). However, PCR-based assays required specialized apparatus, and these requirements rendered PCR-based techniques unsuitable for use in field, point-of-care and resource-poor settings (Jodi Woan-Fei et al., 2015; Wang et al., 2015c, 2016c). Hence, a simple and specific assay for rapid diagnosis of the two pathogens is extremely important to institute adequate antimicrobial therapy, facilitate clinical care, infection control, and epidemiologic investigations.

The increasing use of molecular diagnostic techniques has emphasized speed and simplicity as key criteria for adoption in point-of-care detection, “on-site” testing, field diagnosis and more, and the isothermal amplification technologies were well-suited for these applications. To date, a wide variety of isothermal amplification methodologies have been devised for use in molecular analyses (Asiello and Baeumner, 2011; Craw and Balachandran, 2012). Loop-mediated isothermal amplification (LAMP), as a reliable, sensitive and rapid detection technique, has been widely applied in many fields, especially in molecular diagnostics (Mori et al., 2013). The analysis of LAMP products has become a major concern, since simplifying detection tools is a major concern. Various monitoring techniques, such as colorimetric agents, turbidimeters, fluorescent agents, gel electrophoresis, lab-on-chip devices and lateral flow biosensors (LFBs), have been employed for analysis of LAMP amplicons (Zhang et al., 2014). In particular, various LFBs have been growingly used as alternative tools for analyzing LAMP products due to their low cost, rapidness, simplicity (Quesada-Gonzalez and Merkoci, 2015). However, the basic mechanism of LAMP-LFB technique did not be expounded in previous reports. Most importantly, these methods were restricted to detecting a single target, limiting wider application of these techniques.

**Figure 1:**
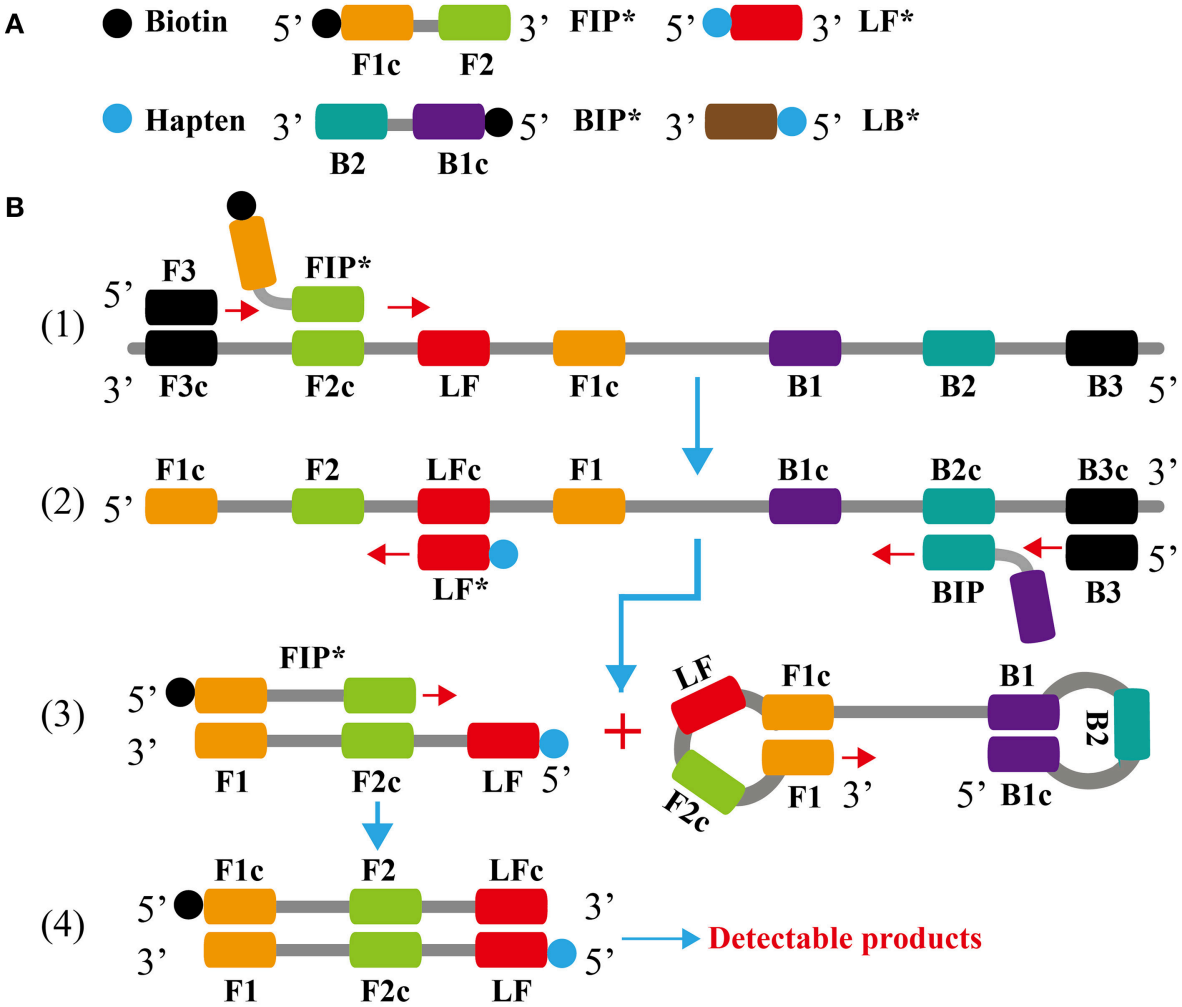
**Outline of loop-mediated isothermal amplification. (A)** Schematic depiction of the new forward/backward inner primer (FIP^*^/BIP^*^) and loop forward/backward primer amplification primer (LF^*^/LB^*^). **(B)** Outline of loop-mediated isothermal amplification with FIP^*^ and LF^*^.

In the current study, we reported on the development of a simple, rapid and sensitive molecular technique, which incorporated LAMP combined with LFB for visual, specific and multiplex detection of nucleic acid sequence. Here, we expounded the basic LAMP-LFB principle (Figure 1) and demonstrated the simplicity and suitability of this assay by simultaneously detecting *E. faecalis* and *S. aureus*. Then, the analytical sensitivity, specificity and practicability of LAMP-LFB assay were determined using pure cultures and spiked blood samples.

## Materials and methods

### Reagents and instruments

The Loopamp kits were purchased from Eiken Chemical. Co., Ltd. (Beijing, China). The visual detection reagent (Hydroxynaphthol blue, HNB) was purchased from BeiJing-HaiTaiZhengYuan Technology Co., Ltd. (Beijing, China). The QIAamp DNA Mini Kit (QIAamp DNA minikits; Qiagen, Hilden, Germany) was purchased from Qiagen. Co., Ltd. (Beijing, China). The Nb.*BsrDI* was purchased from New England Biolabs (Beijing, China). The backing card, sample pad, conjugate pad, nitrocellulose membrane (NC), and absorbent pad were purchased from the Jie Yi Biotechnology. Co., Ltd. (Shanghai, China). The streptavidin-immobilized 30 nm gold nanoparticles (SA-G) was purchased from the Resenbio. Co., Ltd. (XiAn, China). Sheep anti-digoxigenin antibody (anti-Dig), rabbit anti-fluorescein antibody (anti-FITC) and biotinylated bovine serum albumin (biotin-BSA) were purchased from the Abcam. Co., Ltd. (Shanghai, China).

### Preparation of gold nanoparticle-based dipstick biosensor

**Figure 2:**
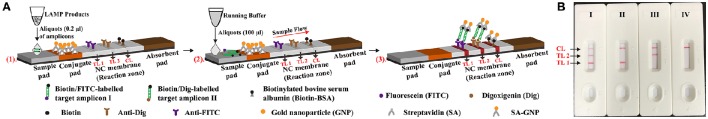
**Schematic illustration of the principle of lateral flow biosensor for visualization of LAMP products. (A)** The test principle of lateral flow biosensor. A1, aliquots (0.2 μl) of LAMP amplicons were deposited on the sample pad of LFB. A2, aliquots (100 μl) of running buffer were also deposited on the sample pad of LFB. A3, The amplicons from target I (*E. faecalis*) were biotin- and fluorescein-labeled, which were captured by the anti-fluorescein body fixed on the first line (test line 1, TL1) of LFB. The amplicons from target II (*S. aureus*) were biotin- and digoxigenin-labeled, which were captured by the anti-digoxigenin body fixed on the second line (test line 2, TL 2) of LFB. The other end of the double-labeled products labeled with biotin could bind streptavidin-conjugated gold nanoparticles for visualization. The excess streptavidin-conjugated gold nanoparticles were captured by biotinylated bovine serum albumin fixed on the third line (control line, CL) of LFB, which demonstrated the working condition of LFB. **(B)** Interpretation of the results. I, a positive result for target I and II (TL1, TL2, and CL area show red bands); II, a positive result for target I (TL1 and CL area show red bands); III, a positive result for target II (TL2 and CL area show red bands); VI, negative (only the control line area shows a red band).

Lateral flow biosensor (4 × 60 mm) was prepared and assembled as previously described with some modifications (Figure 2) (Wang et al., 2016a, 2017). Briefly, the biosensor consisted of four components (the sample pad, conjugate pad, NC membrane and absorbent pad), and they were orderly assembled together on a backing card overlapping 2 mm among them. The capture reagents, including anti-FITC (0.15 mg/ml), anti-Dig (0.2 mg/ml) and biotin-BSA (2.5 mg/ml) in 0.01M phosphate-buffered saline (PBS, PH 7.4), were immobilized by physical adsorption on the reaction regions of the biosensor. On the NC membrane, there are zones as the test line I (conjugated with anti-FITC), test line II (conjugated with anti-Dig) and control line (conjugated with biotin-BSA), with each line separated by 5 mm. SA-G in 0.01M PBS (PH 7.4) was impregnated on the conjugate pad of the biosensor. Then, the whole assembled cards were cut at 0.4 cm widths by a card cutter (Deli No. 8012) and were packaged in a plastic box containing a desiccant gel. All biosensors were stored at the room temperature until use.

### Design of the lamp primers

Based on the *Ef0027* gene (GenBank accession: 1198935) of *E. faecalis* and *nuc* gene (GenBank accession: EF529597) of *S. aureus*, two sets of LAMP primers were designed according to the mechanism of LAMP-LFB technique by using PrimerExplorer V4 (Eiken Chemical) (Brakstad et al., 1992; Liu et al., 2005). The specificity of two sets of LAMP primers was checked using the basic local alignment search tool (BLAST). The details of primers, primer design, locations and modifications were shown in Figure S1 and Table 1. All primers were synthesized by TsingKe. Co., Ltd. (Beijing, China) at HPLC purification grade.

**Table 1:** **The sequences and modifications of the LAMP primers used in this study**.

**Primers name^a^**	**Sequences (5′-3′) and modifications**	**Length^b^**	**Genes**
Ef-F3	ACAGAAAGCGATAGTCGTAGT	21 nt	
Ef-B3	CCTAAAAATGTTAGCTTTCGTGC	23 nt	
Ef-FIP	TGGCTTCATCCATTTGTTGAAAACTTTTCAAGCTATTACGCAACAGT	47 mer	
Ef-FIP^*^	Biotin-TGGCTTCATCCATTTGTTGAAAACTTTTCAAGCTATTACGCAACAGT	47 mer	
Ef-EFIP	HEX-TGCAATG-TGGCTT(BHQ-1)CATCCATTTGTTGAAAACTTTTCAAGCTATTACGCAACAGT	53 mer	*Ef0027*
Ef-BIP	AAGTCGCGGAAATGCTTAAAATG-GTACAAATAGGAAAACTGCCAC	46 mer	
Ef-LF	ATCTTGCACATTGGCAATCA	20 nt	
Ef-LF^*^	FITC-ATCTTGCACATTGGCAATCA	20 nt	
Ef-LB	CGATTGAAGTGTTCGGTGT	19 nt	
Sau-F3	ATGCAAAGAAAATTGAAGTCGA	22 nt	
Sau-B3	GCGTTGTCTTCGCTCCAAAT	20 nt	
Sau-FIP	CGTTTACCATTTTTCCATCAGCATAGTTTGACAAAGGTCAAAGAACT	47 mer	
Sau-FIP^*^	Biotin-CGTTTACCATTTTTCCATCAGCATAGTTTGACAAAGGTCAAAGAACT	47 mer	
Sau-EFIP	Cy5-TGCAATG-CGTT(BHQ2)TACCATTTTTCCATCAGCATAGTTTGACAAAGGTCAAAGAACT	53 mer	*Nuc*
Sau-BIP	TCAAGGCTTGGCTAAAGTTGCTTATTTTCGCTTGTGCTTCACTT	44 mer	
Sau-LF	TACGCTAAGCCACGTCCATA	20 nt	
Sau-LF^*^	Dig-TACGCTAAGCCACGTCCATA	20 nt	
Sau-LB	CCTAACAATACACATGAACAAC	22 nt	

a Ef, E. faecalis; Sau, S. aureus. F3, Forward outer primer; FIP, Forward inner primer; LF, Forward loop primer; LB, Backward loop primer; BIP, Backward inner primer; B3, Backward outer primer; Ef-FIP^*^, 5'-labeled with biotin when used in LAMP-LFB assay; Ef-LF^*^, 5'-labeled with FITC when used in LAMP-LFB assay; Sau-FIP^*^, 5'-labeled with biotin when used in LAMP-LFB assay; Sau-LF^*^, 5'-labeled with Dig when used in LAMP-LFB assay.

b *mer: monomeric unit; nt: nucleotide*.

### Bacterial strains and genomic DNA preparation

A total of 117 strains, which were collected from clinical, food and environmental samples, were used in this study (Table 2). These strains were stored in 10% (w/v) glycerol broth at −70°C and then were subcultured three times on nutrient agar plate at 37°C. The genomic DNA templates were extracted from all culture strains using DNA extraction kits (QIAamp DNA minikits; Qiagen, Hilden, Germany) according to the manufacturer's instructions. The extracted templates were tested with ultraviolet spectrophotometer (Nano drop ND-1000, Calibre, Beijing, China) at A260/280. The templates were stored under at −20°C before the templates were used. The strains of *E. faecalis* (ATCC 51299) and *S. aureus* (ICDC Sau-001) were used for confirmation performance, optimal temperature, sensitivity analysis and practical application conducted in this study. The genomic DNA templates of *E. faecalis* ATCC 51299 and *S. aureus* ICDC Sau-001 were serially diluted 10-fold (2.5 ng, 250 pg, 25 pg, 2.5 pg, 250 fg, 25 fg, and 2.5 fg) for sensitivity analysis of singlex and multiplex LAMP-LFB detections.

**Table 2:** **Bacteria strains used in the study**.

**Bacteria**	**Serovar^a^**	**Strain no. (source of strain)^b^**	**No. of strains**
*Enterococcus faecalis*	U	ATCC 51299	1
	U	Isolated strains (ICDC)	29
*Staphylococcus aureus*	U	ICDC-NPSau001	1
	U	ICDC-NPSau002	1
	U	ICDC-NPSau003	1
	U	ICDC-NPSau004	1
	U	ICDC-NPSau005	1
	U	Isolated strains (ICDC)	25
*Enterococcus faecium*	U	ATCC BAA340	1
	U	Isolated strains (ICDC)	10
*Staphylococcus epidermidis*	U	Isolated strains (ICDC)	5
*Staphylococcus saparophytics*	U	Isolated strains (ICDC)	3
*Enterococcus hirae*	U	Isolated strains (ICDC)	1
*Streptococcus suis*	U	Isolated strains (ICDC)	1
*Streptococcus pneumoniae*	19A	ATCC 700674	1
	14	ATCC 6314	1
*Aeromonas hydrophila*	U	Isolated strains (ICDC)	1
*Klebsiella pneumoniae*	U	Isolated strains (ICDC)	1
*Enteropathogenic E. coli*	U	Isolated strains (ICDC)	1
*Enterotoxigenic E. coli*	U	Isolated strains (ICDC)	1
*Enteroaggregative E. coli*	U	Isolated strains (ICDC)	1
*Enteroinvasive E. coli*	U	Isolated strains (ICDC)	1
*Enterohemorrhagic E. coli*	U	Isolated strains (ICDC)	1
*Acinetobacter baumannii*	U	Isolated strains (ICDC)	1
*Streptococcus sanguis*	U	Isolated strains (ICDC)	1
*Streptococcus bovis*	U	Isolated strains (ICDC)	1
*Pseudomonas aeruginosa*	U	Isolated strains (ICDC)	1
*Campylobacter jejuni*	U	Isolated strains (ICDC)	1
*Bntorobater sakazakii*	U	Isolated strains (ICDC)	1
*Citro freumdii*	U	Isolated strains (ICDC)	1
*Bacillus cereus*	U	Isolated strains (ICDC)	1
*Vibrio cholerae*	U	Isolated strains (ICDC)	1
*Vibrio parahaemolyticus*	U	Isolated strains (ICDC)	1
*Vibrio vulnificus*	U	Isolated strains (ICDC)	1
*Yersinia enterocolitica*	U	ATCC 23715	1
*Enterobacter cloacae*	U	Isolated strains (ICDC)	1
*Salmonella enteric*	U	Isolated strains (ICDC)	1
*Shigella flexneri*	1d	ICDCNPS001	1
*Shigella boydii*	U	Isolated strains (ICDC)	1
*Listeria monocytogenes*	4a	ATCC 19114	1
	4c	ATCC 19116	1
	4d	ATCC 19117	1
	4e	ATCC 19118	1
	4b	ICDC LM419	1
*Listeria innocua*	U	ATCC BAA-680	1
*Listeria grayi*	U	ATCC 25402	1
*Listeria selligeri*	U	ATCC 35967	1
*Listerai welshimeri*	U	ATCC 35897	1
*Listeria ivanovii*	U	ATCC BAA-678	1
*Plesiomonas shigelloides*	U	ATCC 51903	1

a *U, unidentified serotype*.

b *ATCC, American Type Culture Collection; ICDC, National Institute for Communicable Disease Control Disease Control and Prevention, Chinese Center for Disease Control and Prevention*.

### The standard *LAMP* assay

In order to examine the availability of two sets of LAMP primers, the singlex LAMP reaction either for *E. faecalis* strains or *S. aureus* strains was carried out as the standard LAMP assay, which has been described in previous report (Wang et al., 2015b). The amplification mixtures (25 μl) consisted of the following: 0.8 μM each FIP^*^ and FIP primers, 1.6 μM BIP primers, 0.8 μM each LF^*^ and LB primer, 0.4 μM each F3 and B3 primers, 12.5 μl 2 × reaction mix, 1 μl (8 U) of *Bst* DNA polymerase and 1 μl DNA template. The mixtures were heated at 62°C for 1 h and then terminated at 85°C for 5 min. Mixtures with 1 μl genomic template of *Listeria monocytogens* strains (*L. monocytogenes*, ATCC19114) and *Shigella* (ICDCNP001) strains were selected as negative controls, and mixtures with 1 μl double distilled water (DW) were used as a blank control. Four determination techniques, including colorimetric indicators (such as HNB reagent), agarose gel electrophoresis, real-time turbidity and LFB analysis, were employed to monitor the LAMP amplifications. Moreover, the multiple endonuclease restriction real-time loop-mediated isothermal amplification (MERT-LAMP) was employed to achieve real time fluorescence measurement of LAMP reaction (Wang et al., 2015a).

Then, we tested the optimal amplification temperature of two sets of LAMP primers, and the LAMP amplifications were performed at a constant temperature according to the standard LAMP reaction. Various amplification temperatures ranging from 60°C to 67°C with 1°C intervals were examined, and the reactions were monitored by using the Loopamp Real-time Turbidimeter LA-320 (Eiken Chemical Co., Ltd, Japan). A positive reaction was defined as a threshold value of >0.1 within 1 h and a mixture without a genomic template was chose as a negative control.

### The multiplex LAMP reaction

To ensure the simultaneous amplification of two sets of LAMP primers in a tube, the MERT-LAMP technology was applied to adjusting the concentration of the primers (Wang et al., 2015a). Briefly, each 25-μl MERT-LAMP mixture contained 0.5 μM Ef-EFIP and Ef-FIP primers, 1 μM Ef-BIP primer, 0.5 μM each Ef-LF and Ef-LB primers, 0.4 μM each Ef-F3 and Ef-B3 primers, 0.8 μM Sau-EFIP and Sau-FIP primer, 1.6 μM Sau-BIP primer, 0.8 μM each Sau-LF and Sau-LB primers, 0.4 μM each Sau-F3 and Sau-B3 primers, 12.5 μl 2 × reaction mix, 1 μl (8 U) of *Bst* DNA polymerase, 1.5 μl (15 U) of Nb.*BsrDI* endonuclease and 1 μl DNA template DNA each of *E. faecalis* strains and *S. aureus* strains. The multiplex MERT-LAMP reactions were conducted at 62°C for 1 h in a real-time system. The mixtures without the genomic DNA template were selected as a negative control. At least two replicates of each dilution were tested to verify the sensitivity of multiplex MERT-LAMP reactions.

On the basis, the multiplex LAMP was performed as the following system: each 25-μl multiplex LAMP mixture contained 0.5 μM Ef-FIP^*^ and Ef-FIP primers, 1 μM Ef-BIP primer, 0.5 μM each Ef-LF^*^ and Ef-LB primers, 0.4 μM each Ef-F3 and Ef-B3 primers, 0.8 μM Sau-FIP^*^ and Sau-FIP primers, 1.6 μM Sau-BIP primer, 0.8 μM each Sau-LF^*^ and Sau-LB primers, 0.4 μM each Sau-F3 and Sau-B3 primers, 12.5 μl 2 × reaction mix, 1 μl (8 U) of *Bst* DNA polymerase, and 1 μl template DNA each of *E. faecalis* strains and *S. aureus* strains. The multiplex LAMP reactions also were conducted at 62°C for 1 h and at least two replicates of each dilution were determined.

### Analytical sensitivity of the LAMP-LFB assay

In order to examine the analytical sensitivity of singlex and multiplex LAMP-LFB assays, genomic DNA templates from pure culture of *E. faecalis* ATCC 51299 and *S. aureus* ICDC Sau-001 were serially diluted 10-fold (2.5 ng, 250 pg, 25 pg, 2.5 pg, 250 fg, 25 fg, and 2.5 fg) for confirming the limit of detection (LoD). The LoD of LAMP-LFB technology was confirmed by DNA amount of the genomic template. Moreover, a comparative analysis of LAMP-LFB, qPCR and PCR techniques was conducted in the study. The *E. faecalis*-qPCR, *E. faecalis*-PCR, *S. aureus*-qPCR, and *S. aureus*-PCR assays have been established in previous studies, which was employed to confirm the LoD of qPCR and PCR methodologies (Santo Domingo et al., 2003; Peters et al., 2007).

### Evaluation of the specificity of the LAMP-LFB assay

To assess the analytical specificity of the LAMP-LFB methodology, the multiplex LAMP reactions were performed under the conditions described above with the genomic DNA templates from 117 strains (Table 2). Analysis of each sample was carried out twice independently. Furthermore, the analytical sensitivity of LAMP-LFB assay was further confirmed using MERT-LAMP technique.

### Practical application of LAMP-LFB to *E. faecalis* and *S. aureus* analysis in blood samples

The human blood samples were acquired from a healthy donor with the written informed consent. Our study was reviewed and approved by the ethics committee of the National Institute for Communicable Disease Control and Prevention, China CDC, according to the medical research regulations of the Ministry of Health China (Approval No. ICDC2014003).

To demonstrate the applicability of LAMP-LFB assay, two strains, including *E. faecalis* (ATCC 51299) and *S. aureus* (ICDC-NPSau001), were applied to test the analytical sensitivity of the LAMP-LFB in blood samples. Two bacteria suspensions (with an optical density at 0.6), which contained target *E. faecalis* and *S. aureus* strains, respectively, were prepared in 1000 μl of sterile saline. The numbers of colony forming units (CFU) in the two suspensions were obtained using a plate-counting technique according to previous study (Harris et al., 2008). Briefly, serial 10-fold dilution (10^−1^–10^−8^) of two suspensions was carried out, and the aliquots of 100 μl appropriate dilution (10^−6^) were spread onto blood agar in triplicate and incubated at 37°C. After 24 h, the numbers of CFU were counted. Then, the aliquots 100 μl of each suspension with appropriate dilutions (10^−3^, 10^−4^, 10^−5^, 10^−6^, 10^−7^, and 10^−8^) were simultaneously added into the blood samples (800 μl), and the numbers of *E. faecalis* were adjusted to approximate 71000 CFU/ml, 7100 CFU/ml, 710 CFU/ml, 71 CFU/ml, 7.1 CFU/ml and 0.71 CFU/ml, and *S. aureus* for 68000 CFU/ml, 6800 CFU/ml, 680 CFU/ml, 68 CFU/ml 6.8 CFU/ml and 0.68 CFU/ml. Aliquots (100 μl) of the artificially contaminated blood samples were subjected to extract genomic templates, which were eluted in 10 μl of Qiagen elution buffer. Aliquots (2 μl) of extracted genomic templates were used for LAMP-LFB, MERT-LAMP, qPCR and PCR tests. Non-contamination blood sample was chosen as negative control. This analysis was independently carried out in twice.

## Results

### Development of the LAMP-LFB assay

In the LAMP system (Figure 1B), the forward inner primer (FIP^*^) initiates LAMP amplification at the F2c site of the target sequence, and the newly synthesized strand is displaced by the upstream synthesis from the forward primer F3 (Step 1). After 3 primers, including loop forward primer (LF^*^), backward inner primer (BIP) and backward primer (B3), anneal to the newly synthesized strand (Step 2). Then, the *Bst* polymerase extends in tandem yielding two distinct products (Step 3). The LF^*^ product acts as the template for further extension by primer FIP^*^, forming a double-labeled detectable product (Step 4). The end of the target amplicon (FIP^*^/LF^*^product) was labeled with biotin, and the other end with hapten. One hapten is assigned to one LAMP primer set, which offer the possibility for multiplex LAMP analysis. The details of the amplification process for BIP product have been descripted in previous reports (Wang et al., 2015a). Moreover, a double-labeled detectable product (BIP^*^/LB^*^), which is similar to the detectable BIP^*^/LF^*^ product, can be produced when the BIP primer is labeled with biotin at the 5′ end and LB primer for hapten. The result readout of LAMP-LFB assay is shown in Figure 2.

### Confirmation and detection of *E. faecalis*- and *S. aureus*-LAMP-LFB products

**Figure 3:**
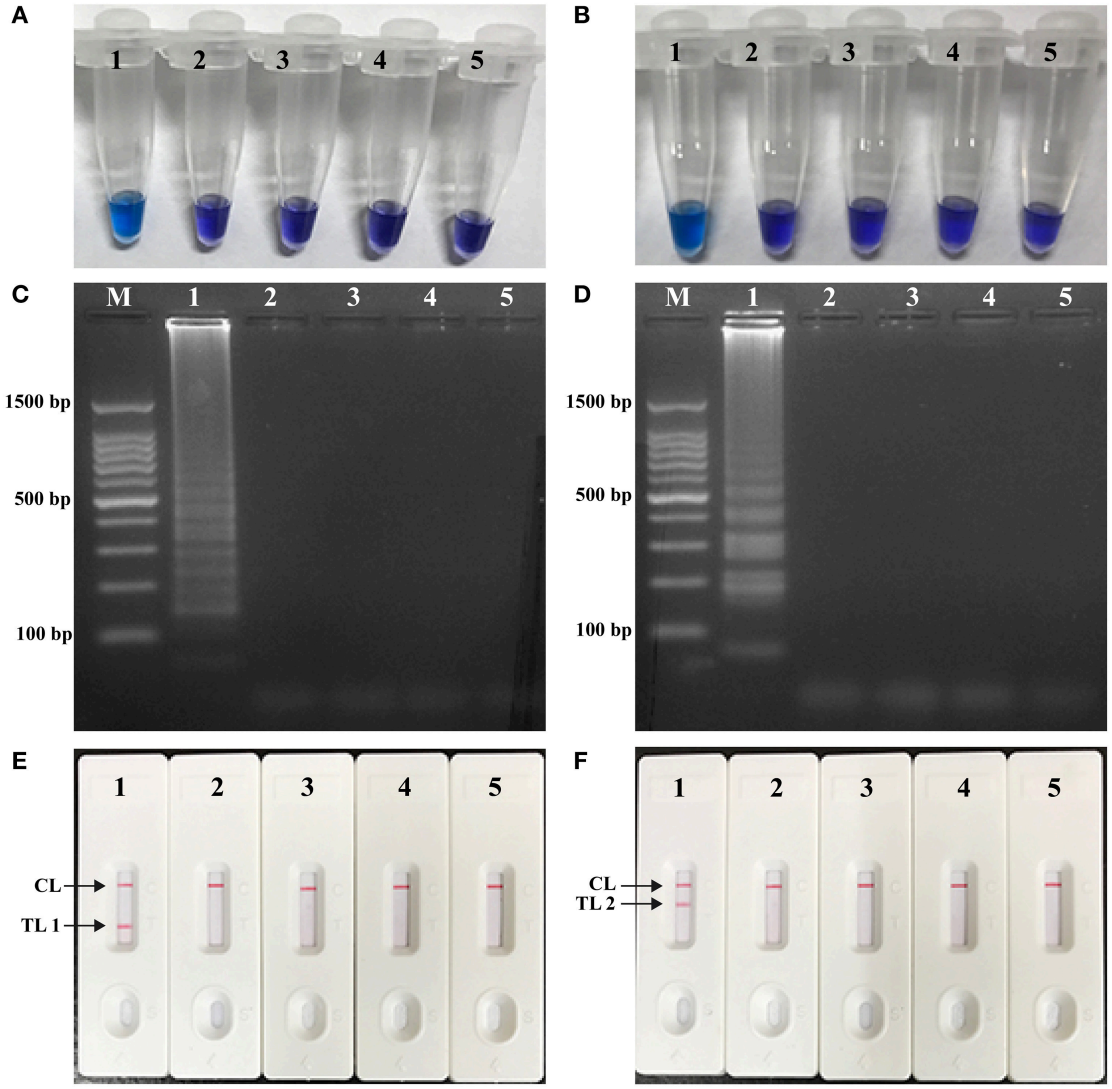
**Detection and confirmation of ***E. faecalis***- and ***S. aureus***-LAMP products. (A,B)** Color change of *E. faecalis*- and *S. aureus*-LAMP tubes; **(C,D)** 2%agarose gel electrophoresis applied to *E. faecalis*- and *S. aureus*-LAMP products; **(E,F)**, LFB applied for visual detection of *E. faecalis*- and *S. aureus*-LAMP products. Tube A1 (lane C1, biosensor E1), positive amplification; tube A2 (lane C2, biosensor E2), negative amplification (*S. aureus*), tube A3 (lane C3, biosensor E3), negative amplification (*L. monocytogenes*), tube A4 (lane C4, biosensor E4), negative amplification (*Shigella*), tube A5 (lane C5, biosensor E5), blank control (DW); Tube B1 (lane D1, biosensor F1), positive amplification; tube B2 (lane D2, biosensor F2), negative amplification (*E. faecalis*), tube B3 (lane D3, biosensor F3), negative amplification (*L. monocytogenes*), tube B4 (lane D4, biosensor F4), negative amplification (*Shigella*), tube B5 (lane D5, biosensor F5), blank control (DW); Lane M, DL 50-bp DNA marker.

In order to verify the availability of two sets of LAMP primers, the LAMP amplification mixtures were carried out in the absence or presence genomic templates according to the standard condition. The color of the positive amplifications in *E. faecalis*- and *S. aureus*-LAMP tubes changed from violet to blue when detected with HNB reagents, whereas the color remained violet in the negative controls and blank tube with 1 h incubation periods (Figures 3A,B). The LAMP products were analyzed by 2% agarose gel electrophoresis, the ladder-liker patterns were observed in the positive reactions but not in negative controls and blank control (Figures 3C,D). Moreover, it was observed that clear visible red bands in positive amplifications were seen for the test line I (TL I for *E. faecalis* detection) and test line II (TL II for *S. aureus* detection), only a red band (control line; CL) was seen in negative and blank controls (Figures 3E,F). These results demonstrated that the two primer sets were available for establishment of LAMP-LFB assay for *E. faecalis* and *S. aureus* detection.

### Optimal amplification temperature of *E. faecalis*- and *S. aureus*-LAMP primers

**Figure 4:**
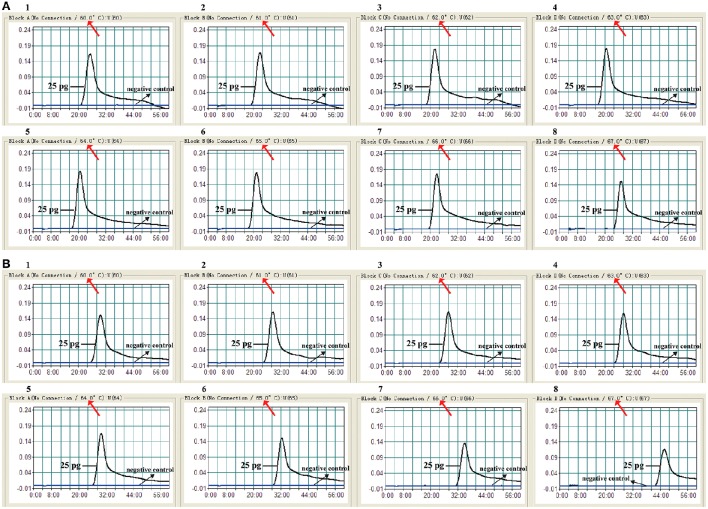
**The optimal amplification temperature for LAMP reactions**. The standard LAMP reactions for detection of *E. faecalis* **(A)** and *S. aureus* **(B)** were monitored by real-time measurement of turbidity and the corresponding curves of concentrations of DNA were marked in the figures. The threshold value was 0.1 and the turbidity of >0.1 was considered to be positive. Eight kinetic graphs (1–8) were generated at various temperatures (60–67°C, 1°C intervals) with target pathogens DNA at the level of 25 pg per reaction. **(A)** The graphs from 2 to 7 showed robust amplification; **(B)** the graphs from 2 to 4 showed robust amplification.

To test the optimal reaction temperature of *E. faecalis*- and *S. aureus* -LAMP primers, the *E. faecalis*- and *S. aureus*-LAMP amplifications were conducted at the different temperature (60°C to 67°C) with 1°C intervals according to the standard condition, and the strains *E. faecalis* ATCC 51299 and *S. aureus* ICDC Sau-001 were selected as the positive control to examine the optimal temperature at the level of 25 pg of each template per tube. The results were analyzed by means of real time turbidity detection. The typical kinetics graphs were generated from the eight temperatures, with the faster reactions observed for approach temperature of 61°C to 66°C for the *E. faecalis*- LAMP reactions and 61°C to 64°C for *S. aureus*-LAMP reactions (Figure 4). The reaction temperature of 62°C was used for the rest of singlex and multiplex-LAMP amplifications carried out in this report.

### Sensitivity of LAMP-LFB detection for a single target

**Figure 5:**
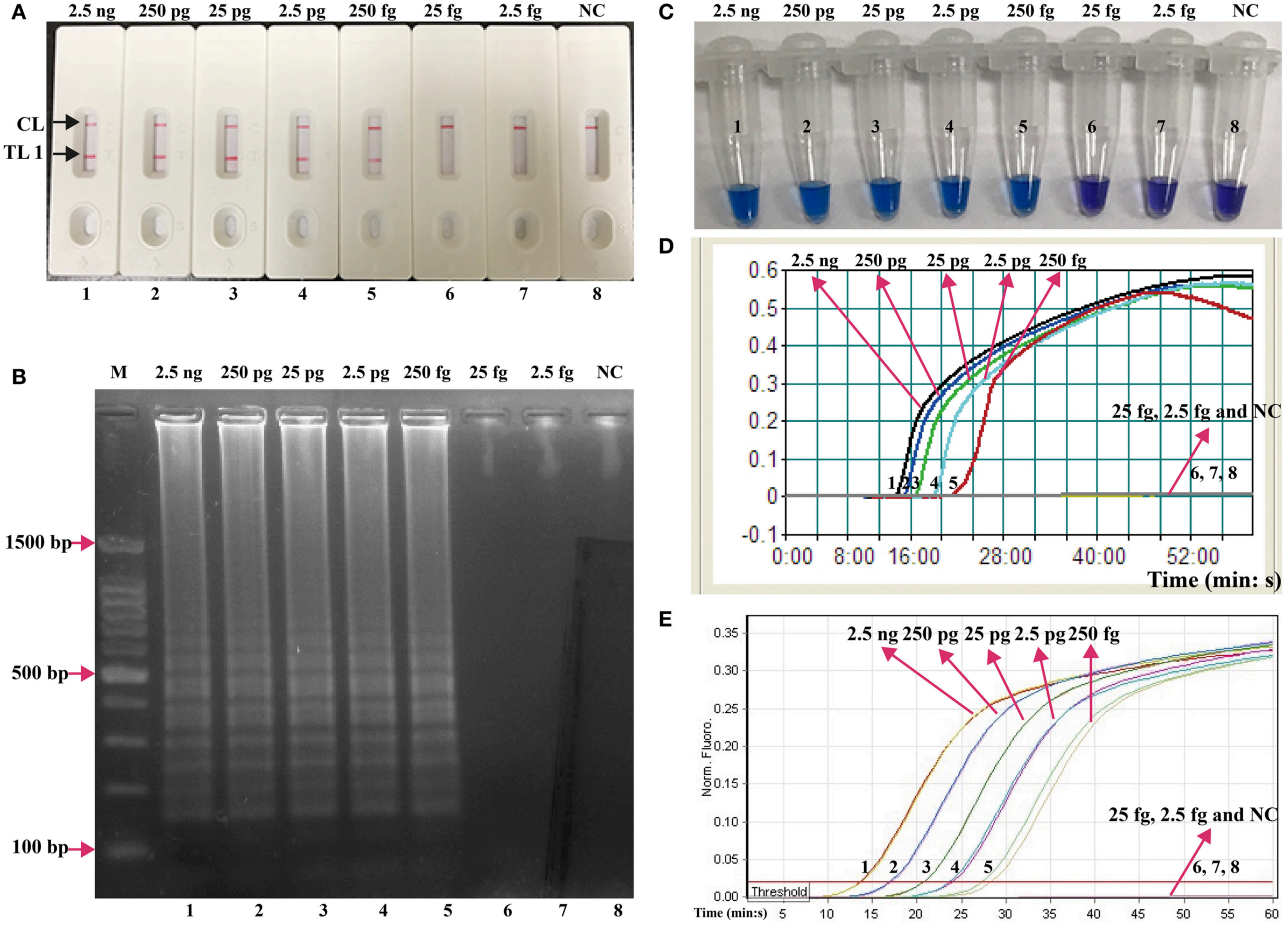
**Analysis of a single target in a standard LAMP reaction**. LAMP primers targeting the *Ef0027* (*E. faecalis*) gene were used in standard reactions and the serial dilutions (2.5 ng, 250 pg, 25 pg, 2.5 pg, 250 fg, 25 fg, and 2.5 fg) of target templates were subjected to LAMP amplification. **(A)** LFB applied for visual detection of *E. faecalis*-LAMP products. **(B)** 2% agarose gel electrophoresis applied to *E. faecalis*-LAMP products. **(C)** HNB applied for visual detection of *E. faecalis*-LAMP products. **(D)** Real-time turbidity applied for detection of *E. faecalis*-LAMP products. **(E)** Real-time fluorescence applied for detection of *E. faecalis*-LAMP products. Biosensors/lanes/tubes/turbidity signals/ fluorescence signals 1-7, *E. faecalis* genomic DNA (2.5 ng-2.5 fg), biosensor/lane/tube/turbidity signal/ fluorescence signal 8, blank control (DW).

**Figure 6:**
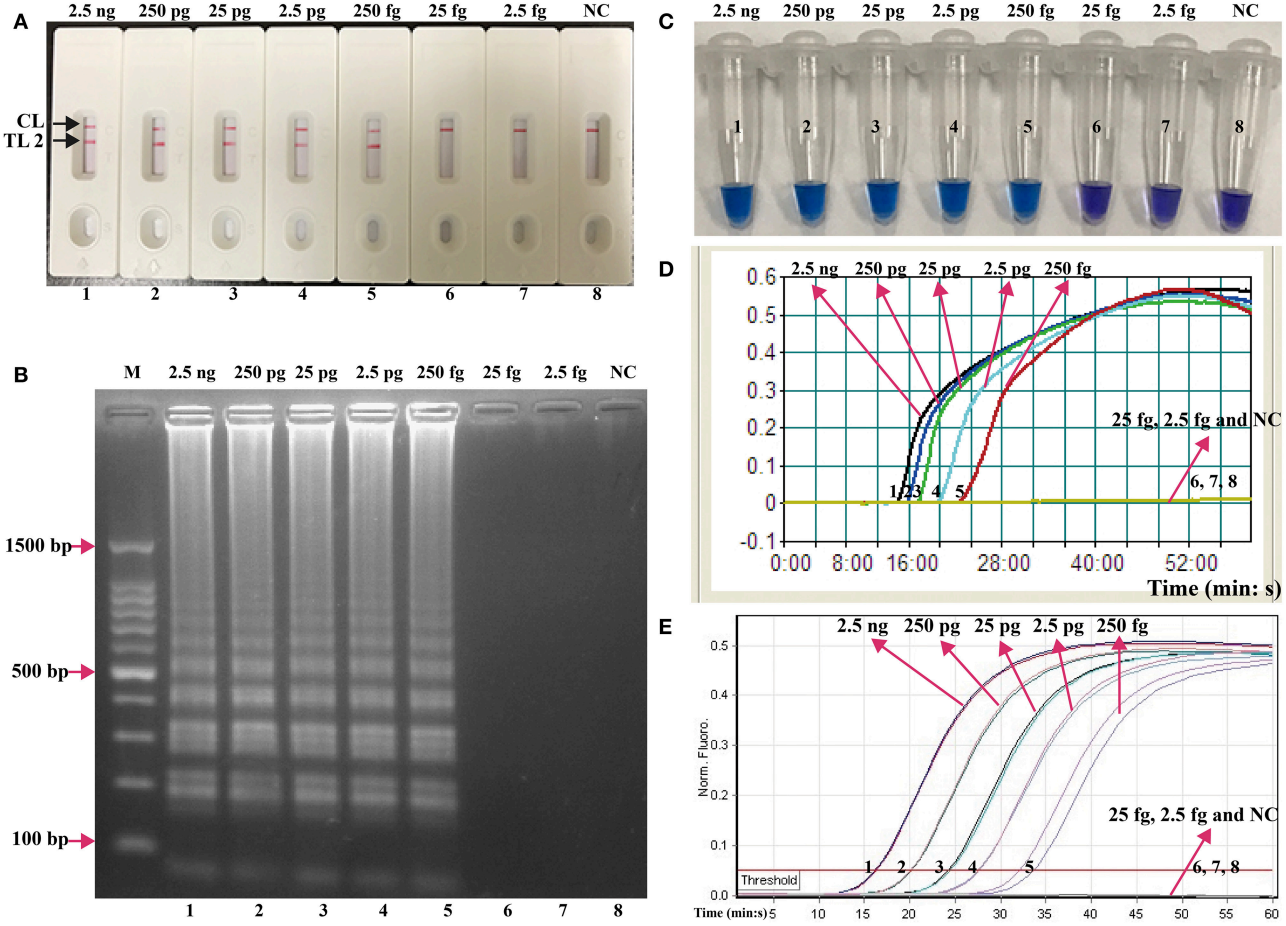
**Analysis of a single target in a standard LAMP reaction**. LAMP primers targeting the *nuc* (*S. aureus*) gene were used in standard reactions and the serial dilutions (2.5 ng, 250 pg, 25 pg, 2.5 pg, 250 fg, 25 fg, and 2.5 fg) of target templates were subjected to LAMP amplification. **(A)** LFB applied for visual detection of *S. aureus*-LAMP products. **(B)** 2%agarose gel electrophoresis applied to *S. aureus*-LAMP products. **(C)** HNB applied for visual detection of *S. aureus*-LAMP products. **(D)** Real-time turbidity applied for detection of *S. aureus*-LAMP products. **(E)** Real-time fluorescence applied for detection of *S. aureus*-LAMP products. Biosensors/lanes/tubes/turbidity signals/ fluorescence signals 1-7, *S. aureus* genomic DNA (2.5 ng-2.5 fg), biosensor/lane/tube/turbidity signal/ fluorescence signal 8, blank control (DW).

The serial dilutions (2.5 ng, 250 pg, 25 pg, 2.5 pg, 250 fg, 25 fg, and 2.5 fg per microliter) of the total genomic DNA were used as templates to determine the analytical sensitivity of *E. faecalis*- and *S. aureus*-LAMP-LFB assays. The limit of detection (LoD) of LAMP-LFB assays for independently detecting *Ef0027* (*E. faecalis*-specific gene) and *nuc* (*S. aureus*-specific gene) sequences was 250 fg of genomic templates per reaction (Figures 5, 6). As expected, the biosensor showed clear visible red bands for both TL I and CL when the products came from positive *E. faecalis*-LAMP reactions, and only the CL was seen for negative *E. faecalis*-LAMP reactions and blank control (Figure 5A). Likewise, the biosensor also displayed clear visible red bands for both TL II and CL when the products came from positive *S. saures*-LAMP reactions, and only the CL was seen for *S. saures*-LAMP reactions and blank control (Figure 6A). The LoD of LAMP-LFB method for independently detecting *Ef0027* gene or *nuc* gene was 250 fg per reaction. The positive *E. feacalis*- and *S. aureus*-LAMP products by 2% agarose gel electrophoresis were observed as the ladder-like patterns but not in negative reactions and blank control, and the LoD of the agarose gel electrophoresis detection for *E. feacalis*- and *S. aureus*-LAMP amplification was consist with biosensor analysis (Figures 5B, 6B).

Three monitoring techniques, including colorimetric indicators (such as HNB reagent), turbidimeters and MERT-LAMP analysis, were applied to further verifying the assay sensitivity. By HNB reagent, a color shift of positive reaction in *E. faecalis*- and *S. aureus*-LAMP tubes was directly seen from violet to blue (Figures 5C, 6C). The *E. faecalis*- and *S. aureus*-LAMP reactions were analyzed by real time measurement of turbidity and the LoD of *E. faecalis*- and *S. aureus*-LAMP method for independently detecting *Ef0027* gene or *nuc* gene was 250 fg per reaction (Figures 5D, 6D). By real time fluorescence analysis, the sensitivity of *E. faecalis*- and *S. aureus*-MERT-LAMP approaches for independently detecting *Ef0027* gene or *nuc* gene was also 250 fg per reaction (Figures 5E, 6E). These results indicated that the assay sensitivity by agarose gel electrophoresis, real time turbidity, HNB reagent, and real time fluorescence detection for *E. faecalis*- and *S. aureus*-LAMP amplifications was in conformity with LFB analysis (Figures 5, 6). Moreover, the LoD of *E. faecalis*- and *S. aureus*-LAMP-LFB assay was 10- and 100-fold more sensitive than that of the qPCR and PCR assays (Figures 5, 6 and Table 3).

**Table 3:** **The sensitivity for singlex LAMP-LFB reaction targeting ***Ef0027*** and ***nuc*** genes compared with that of qPCR and conventional PCR assays**.

**Approaches^a^**	**Isothermal amplification**	**Regions recognized**	**Multiplex detection**	**LoD for ***E. faecalis/S. aureus*** (no./reaction)^b^**
LAMP-LFB	+	8	+	250 fg/250 fg
qPCR	−	3	+	2.5 pg/2.5 pg
PCR	−	2	+	25 pg/25 pg

a *LAMP, loop-mediated isothermal amplification; LFB, lateral flow biosensor; qPCR, quantitative real-time PCR*.

b *LoD, limit of detection*.

### Sensitivity of LAMP-LFB detection of multiple targets in a reaction

According to multiplex MERT-LAMP reaction, the concentration of two sets of LAMP primers was adjusted to facilitate the simultaneous amplification in a vessel and guarantee the generation of reliable multiplex LAMP products. Two different fluorescence curves were simultaneously yielded from multiplex MERT-LAMP reactions containing two complete MERT-LAMP primer sets and their corresponding genomic templates (Figure S2). Hence, the multiplex MERT-LAMP system was able to simultaneously amplify and correctly identify *Ef0027* gene and *nuc* gene in a single tube.

**Figure 7:**
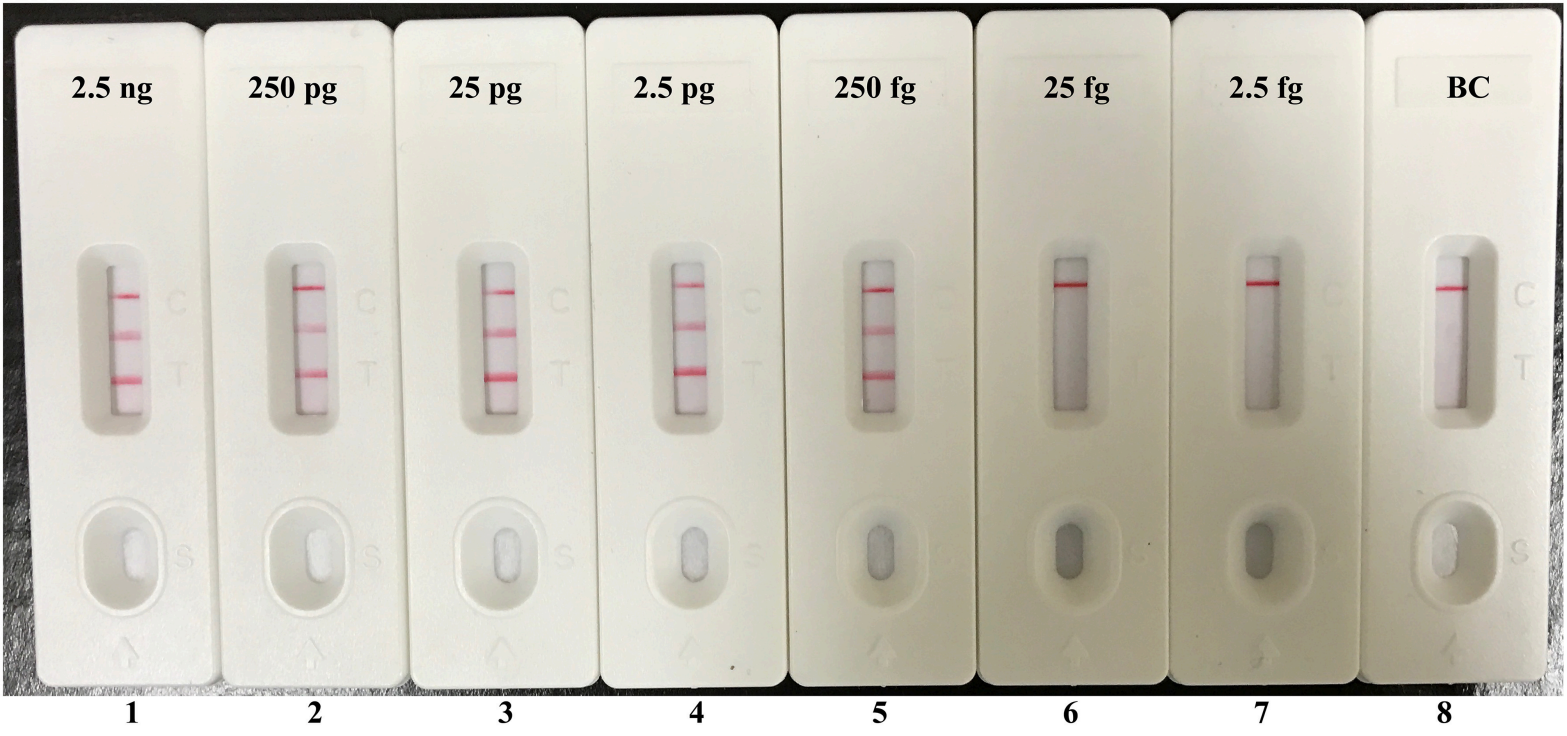
**Visual detection of multiplex targets in a multiplex LAMP reaction**. Two sets of MCDA primers targeting *Ef0027* and *nuc* genes were simultaneously added to a reaction tube and the sensitivity of multiplex LAMP for simultaneously detecting *E. faecalis* and *S. aureus* was analyzed by visual format. Biosensors 1, 2, 3, 4, 5, 6, 7, and 8 represent DNA levels of 2.5 ng (each of *E. faecalis* and *S. aureus* templates), 250 pg (each of *E. faecalis* and *S. aureus* templates), 25 pg (each of *E. faecalis* and *S. aureus* templates), 2.5 pg (each of *E. faecalis* and *S. aureus* templates), 250 fg (each of *E. faecalis* and *S. aureus* templates), 25 fg (each of *E. faecalis* and *S. aureus* templates), 2.5 fg (each of *E. faecalis* and *S. aureus* templates) and blank control (DW). The LoD of multiplex LAMP-LFB assay for *E. faecalis* and *S. aureus* detection was 250 fg per reaction.

Instead of Ef-EFIP, Ef-LF, Sau-EFIP, and Sau-LF primers in multiplex MERT-LAMP system, the Ef-FIP^*^, Ef-LF^*^, Sau-FIP^*^, and Sau-LF^*^ primers were added into the multiplex LAMP amplification mixture for replicating all possible target sequences and labeling all the possible target amplicons. After multiplex LAMP, the reaction mixtures were directly detected using the biosensor. Three visible red lines, including TL I, TL II, and CL, appeared on the biosensor, suggesting positive results for two target organisms, whereas only a visible band at the control zone appeared, suggesting negative results at the concentrations lower than 250 fg of DNA templates per vessel and blank control (Figure 7). Therefore, the assay sensitivity of multiplex LAMP-LFB technique for simultaneously identifying *Ef0027* and *nuc* genes was also 250 fg of DNA templates per reaction, which was in complete accordance with the singlex *E. faecalis*- and *S. aureus*-LAMP-LFB detection.

### The optimal duration of time require for LAMP-LFB assay

On the basis of multiplex LAMP-LFB method, we examined the optimal duration of time require for the multiplex LAMP-LFB detection during the amplification stage, and six distinct reaction times (10, 20, 30, 40, 50, and 60 min) were compared at 62°C. The lowest DNA levels (250 fg of *E. faecalis* and *S. aureus* templates) exhibited three visible red lines when the reaction lasted for 40 min at 62°C, thus a amplification time of 40 min was recommended as the optimal time for the LAMP-LFB method during the amplification stage (Table 4). Herein, the whole process, including sample (such as blood sample) processing (30 min), amplification (40 min) and result indicating (2 min), could be finished within 75 min.

**Table 4:** **Optimal duration of time required for the multiplex LAMP-LFB assay^a^**.

**Time/min**	**DNA dilution for** ***E. faecalis/S. aureus*** **(no./μl)**							
	**2.5 ng**	**250 pg**	**25 pg**	**2.5 pg**	**250 fg**	**25 fg**	**2.5 fg**	**BC^c^**
10	−/−	−/−	−/−	−/−	−/−	−/−	−/−	−/−
20	+/+	+/+	+/+	−/−	−/−	−/−	−/−	−/−
30	+/+	+/+	+/+	+/+	+/+^b^	−/−	−/−	−/−
40	+/+	+/+	+/+	+/+	+/+	−/−	−/−	−/−
50	+/+	+/+	+/+	+/+	+/+	−/−	−/−	−/−
60	+/+	+/+	+/+	+/+	+/+	−/−	−/−	−/−

a *Three replications were performed for each trial*.

b *The result was weakly positive*.

c *BC, blank control*.

### Analytical specificity of multiplex LAMP-LFB assay

**Figure 8:**
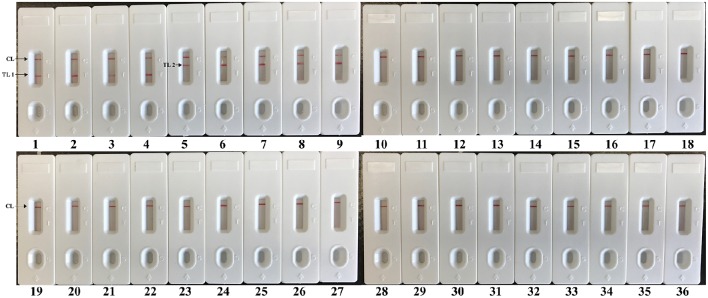
**The specificity of multiplex LAMP assay for different strains**. The multiplex LAMP reactions were conducted using different genomic DNA templates and were monitored by means of visual format. Biosensors 1-3, strains of *E. faecalis* (ATCC 51299), *E. faecalis* (ICDC-NPEf001), *E. faecalis* (ICDC-NPEf002); biosensors 4-9, *S. aureas* (ICDC-NPSau001), *S. aureas* (ICDC-NPSau002), *S. aureas* (ICDC-NPSau003), *S. aureas* (ICDC-NPSau004), *S. aureas* (ICDC-NPSau005), *S. aureas* (Isolated strain); biosensors 13-35, non-*S. aureas*, non-*E. faecalis* strains of *Enterococcus faecium* (ATCC BAA340), *Staphylococcus epidermidis, Staphylococcus saparophytics, Enterococcus hirae, Streptococcus suis, Streptococcus pneumonia, Aeromonas hydrophila, Klebsiella pneumonia, Enteroinvasive E. coli, Enteropathogenic E. coli, Enterotoxigenic E. coli, Enteroaggregative E. coli, Enterohemorrhagic E. coli, Acinetobacter baumannii, Streptococcus sanguis, Streptococcus bovis, Pseudomonas aeruginosa, Campylobacter jejuni, Bntorobater sakazakii, Citro freumdii, Bacillus cereus, Shigella flexneri* and *Listeria monocytogenes*; biosensor 36, blank control.

The assay specificity of the LAMP-LFB assay was evaluated using extracted genomic templates (Roughly 3 ng of genomic templates for each pathogen) from 30 *E. faecalis*, 30 *S. aureus*, 57 non-*E. faecalis* and non-*S. aureus* strains (Table 2). The positive results were specifically yielded only when genomic DNAs of *E. faecalis* and *S. aureus* strains were used as templates. As shown in Figure 8, two red lines (TL I and CL) appeared on the strip, suggesting positive results for all *E. faecalis* strains, whereas TL II and CL appeared on the biosensor, indicating positive results for all *S. aureus* strains. Only a visible red line (CL) at the control zone appeared, suggesting negative results for non-*E. faecalis*, non-*S. aureus* strains and blank control. The multiplex LAMP-LFB assay established here was able to correctly distinguish and simultaneously detect two target pathogens in a reaction. Furthermore, the analytical specificity of multiplex LAMP-LFB assay was further demonstrated using the multiplex MERT-LAMP technique (Figure S3). Hence, these results suggested that the multiplex LAMP-LFB approach described here was feasible to target sequence detection and differentiation.

### Examination of LAMP-LFB assay for artificially contaminated blood sample

**Figure 9:**
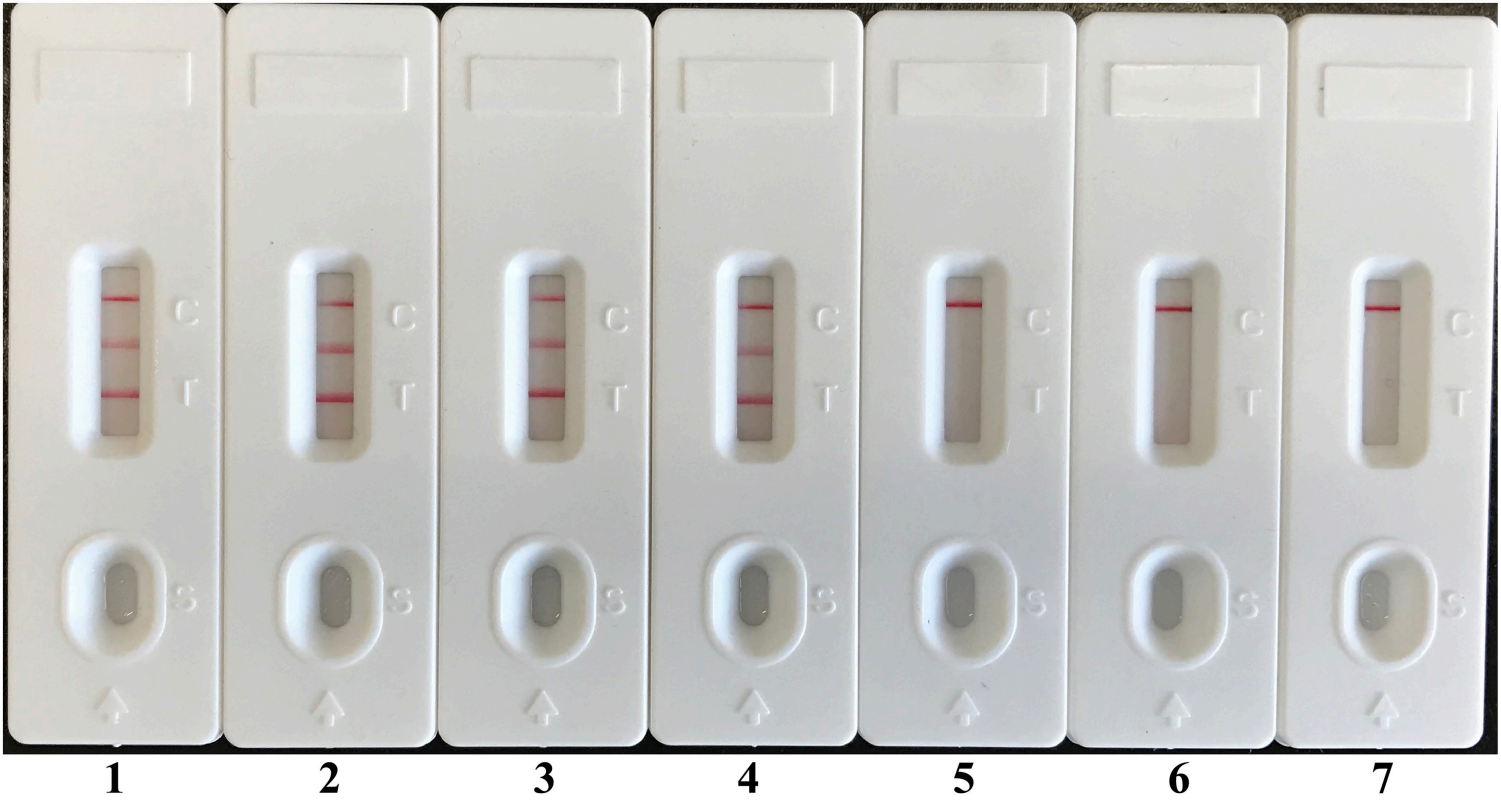
**Analytical sensitivity of LAMP-LFB for simultaneously detecting two target pathogens in artificially contaminated blood samples**. Two sets of LAMP primers targeting *Ef0027* and *nuc* genes were simultaneously added to a reaction vessel. Sensitivity of LAMP-LFB assay for simultaneously detecting *E. faecalis* and *S. aureus* in artificially contaminates blood samples was analyzed by visual format. The biosensors 1, 2, 3, 4, 5, and 6 represent *E. faecalis* DNA levels of 14200 CFU, 1420 CFU, 142 CFU, 14.2 CFU, 1.42 CFU, and 0.142 CFU per reaction; *S. aureus* DNA levels for 13600 CFU, 1360 CFU, 136 CFU, 13.6 CFU, 1.36 CFU, and 0.136 CFU per reaction, and the biosensor 7 represent negative control (non-contaminated blood samples). The LoD of LAMP-LFB method for *E. faecalis* analysis in artificially contaminates blood samples was 14.2 CFU per reaction, and the LoD of LAMP-LFB for *S. aureus* detection in artificially contaminates blood samples was 13.6 CFU per reaction.

In order to demonstrate the usability of LAMP-LFB assay as a detection tool for diagnosing *E. faecalis* and *S. aureus*, the LAMP-LFB method was evaluated by the artificially inoculating *E. faecalis* and *S. aureus* strain into the blood samples. The multiplex LAMP-LFB method generated positive results when the contaminated numbers of *E. faecalis* and *S. aureus* more than 710 CFU/ml (~14.2 CFU/reaction) and 680 CFU/ml (~13.6 CFU/reaction), respectively, and the target pathogens could be simultaneously detected and correctly distinguished in a blood sample (Figure 9, Table 5). The non-contaminated blood sample was seen to be negative.

**Table 5:** **Comparison of LMAP-LFB, qPCR and PCR assay for detection of ***E. faecalis*** and ***S. aureus*** in artificially contaminated blood samples**.

**Detection methods^a^**	**Multiplex detection**	**LoD (no./reaction)**	
		***E. faecalis*** **detection**	***S. aureus*** **detection**
LAMP-LFB	+	14.2 CFU ~ (7.1 × 10^2^ CFU/ml)	13.6 CFU ~ (6.8 × 10^2^ CFU/ml)
qPCR	−	142 CFU ~ (7.1 × 10^3^ CFU/ml)	136 CFU ~ (6.8 × 10^3^ CFU/ml)
PCR	−	1420 CFU ~ (7.1 × 10^4^ CFU/ml)	1360 CFU ~ (6.8 × 10^4^ CFU/ml)

a *LAMP, loop-mediated isothermal amplification; LFB, lateral flow biosensor; qPCR, quantitative real-time PCR*.

The LoD of multiplex LAMP-LFB methodology was identical with that of multiplex MERT-LAMP detection for *E. faecalis* or *S. aureus* in artificially contaminated blood sample (Figure S4, Table 5). Comparatively, the analytical sensitivity of multiplex LAMP-LFB technique was 10- and 100-fold more sensitivity than that of qPCR and PCR methods, respectively (Table 5). The qPCR technique yielded positive results when the contaminate numbers of *E. faecalis* and *S. aureus* amounted to more than 7100 CFU/ml (~142 CFU/reaction) and 6800 CFU/ml (~136 CFU/reaction), conventional PCR assay for 71000 CFU/ml (~1420 CFU/reaction) and 68000 CFU/ml (~1360 CFU/reaction), respectively.

## Discussion

*E. faecalis* and *S. aureus* are two important bacterial pathogens in nosocomial infections, such as surgical site, urinary tract and bloodstream infections (Filetoth, 2008). Thus, rapid, sensitive and reliable detection of *E. faecalis* and *S. aureus* is essential in decreasing the risk factor caused by the two pathogens. This report describes a sensitive LAMP-LFB method for specific detection and accurate identification of the two target organisms in a single assay. Comparing with the existent PCR-based methodologies, the LAMP-LFB method was conducted at a constant temperature during the amplification stage, eliminating the use of a costly specialized apparatus, and only a simple water bath or heat blocker was needed to perform the reaction. Herein, the LAMP-LFB assay established here has potential for “on-site” detection, field test, point-of-care diagnosis and more.

The LAMP-LFB method only required an incubation time of 40 min during the reaction stage (Table 4). The amplification products were directly detected using the biosensor, and result indicating could be finished within 2 min. Analysis of LAMP products with a lateral flow biosensor is not only speedy, but also less error-prone and simpler than analysis by the other monitoring techniques (colorimetric indicators, agarose gel electrophoresis, real-time turbidity and fluorescence measurement) employed in the current report (Figures 3, 5, 6). Consequently, the whole procedure, including specimen processing (30 min), incubation (40 min) and analysis (2 min), could be competed with 75 min. Thus, the rapid identification of target pathogens was valuable for determining the choice of rational therapy in clinical laboratories.

Although, many previous studies have applied LFB for the analysis of LAMP amplicons, the basic mechanism did not be depicted (Kiatpathomchai et al., 2008; Njiru, 2012; Kaewphinit et al., 2013; Yin et al., 2016). To the best of our knowledge, this is first report expounding the basic LAMP-LFB principle and providing the details on biosensor detection (Figures 1, 2). In the LAMP-LFB system, FIP^*^ or BIP^*^ primer, which involved in isothermal amplification, was labeled with biotin at the 5′ end. LF^*^ or LB^*^ primer was labeled with hapten at the 5′ end. During the reaction stage, the double-labeled detectable products were formed, which were yielded from biotin-labeled FIP^*^ (BIP^*^) primers and hapten-labeled LF^*^ (LB^*^) primers. One hapten was assigned to one target primer set, thus LAMP-LFB method permitted detection of multiple nucleic acid sequences in a single reaction using the FIP^*^(BIP^*^) and LF^*^(LB^*^) primers. The amplicons from target I (*E. faecalis*) were biotin- and fluorescein-labeled, which derived from biotin-labeled FIP^*^ primer and fluorescein-labeled LF^*^ primer, and were captured by the anti-fluorescein body fixed on the first line of LFB, known as the test line I. The amplicons from target II (*S. aureus*) were biotin- and digoxigenin-labeled, which derived from biotin-labeled FIP^*^ primer and digoxigenin-labeled LF^*^ primer, and were captured by the anti-digoxigenin body fixed on the second line of LFB, known as the test line II. The other end of the double-labeled products labeled with biotin could bind streptavidin-conjugated gold nanoparticles for visualization. The excess streptavidin-conjugated gold nanoparticles were captured by biotinylated bovine serum albumin fixed on the third line of LFB, known as the control line, which demonstrated the working condition of LFB.

Two species-specific genes (*Ef0027* and *nuc*) present in both *E. faecalis* and *S. aureus* were selected as target genes for designing the LAMP-LFB primers (Brakstad et al., 1992; Liu et al., 2005). A total of 117 strains (30 *E. faecalis*, 30 *S. aureus*, 57 non-*E. faecalis* and non-*S. aureus* strains) were applied to determining the assay specificity, and the LAMP-LFB assay achieved 100% inclusivity and 100% exclusivity. Positive reactions were seen in the assay of *E. faecalis* strains and *S. aureus* strains, but not for non-*E. faecalis* strains and non-*S. aureus* strains. Moreover, the LAMP-LFB assay described here could simultaneously diagnose and correctly differentiate the two pathogens in a sample, which was conducted at a fixed temperature (62°C) with easily interpretable results (Figure 8). These results indicated that the LAMP-LFB technology developed here was specific to *E. faecalis* and *S. aureus* detection.

In additional to its high specificity, the analytical sensitivity of LAMP-LFB method was assessed using serial dilution of genomic templates. We examined the LAMP-LFB assay in a single target format by using separate amplification of *Ef0027* (*E. faecalis-*specific gene) and *nuc* (*S. aureus*-specific gene) from *E. faecalis* and *S. aureus* genomic templates. The analytical sensitivity of LAMP-LFB technique for independently analyzing *Ef0027* gene or *nuc* gene was 250 fg genomic DNA templates per tube, which was 10- and 100-fold more sensitive than that of the qPCR and PCR approaches (Figures 5, 6 and Table 3). Moreover, the multiplex LAMP-LFB method provided the robust data of analytical sensitivity, which was similar to singlex LAMP-LFB reaction (Figures 5–7). The analytical sensitivity of multiplex LAMP-LFB to simultaneously detect *Ef0027* and *nuc* genes was 250 fg of genomic DNA templates each reaction, which was in conformity with that of singlex LAMP-LFB detections. Although, the LAMP products could be analyzed equally with other four techniques employed in the current study, lateral flow biosensor is likely the preferred technique as the indicating the results is less subjective and does not rely on expensive reagents and complicated instrumentation.

In spiked blood samples, the LoD of 14.2 *E. faecalis* CFU/reaction and 13.6 *S. aureus* CFU/reaction was found for multiplex LAMP-LFB assays, which translated to 710 *E. faecalis* CFU/ml and 680 *S. aureus* CFU/ml of blood sample, respectively (Figure 9, Table 5). In contrast, the detection limit of qPCR approaches was 142 *E. faecalis* CFU/reaction (~7.1 × 10^3^ CFU/ml) and 136 *S. aureus* CFU/reaction (~6.8 × 10^3^ CFU/ml), and conventional PCR methods for 1420 *E. faecalis* CFU/reaction (~7.1 × 10^4^ CFU/ml) and 1360 *S. aureus* CFU/reaction (~6.8 × 10^4^ CFU/ml), suggesting that multiplex LAMP-LFB methodologies were 10- and 100-fold more sensitive than qPCR and PCR technologies for detection of two target pathogens in spiked blood samples.

In conclusion, a LAMP-LFB assay was successfully established for detection of *E. faecalis* and *S. aureus*, causing nosocomial infection, such as bloodstream, surgical site and urinary tract infections. LAMP-LFB assay is simple, sensitive and specific, allowing multiplex and visual analysis of multiple targets in a single reaction. The use of LFB offers a rapid, objective and easily interpretable readout of the assay's results, which eliminates the use of expensive reagents and special apparatus. Thus, the LAMP-LFB technique is a valuable tool for identifying target pathogens in clinical, point-of-care and resource-poor settings. Moreover, the proof-of-concept strategy (LAMP-LFB) may be reconfigured to detect various biomarkers by re-designing the specific LAMP primers.

## Author contributions

YiW, JX, and CY conceived and designed the experiments; YiW, HL, YaW, and LZ performed the experiments; YiW, and HL analyzed the data; YiW, HL, YaW, and LZ contributed reagents/materials/analysis tools; YiW, performed the software; YiW, JX, and CY wrote the paper.

## Funding

This work was supported by grants (Mega Project of Research on the Prevention and Control of HIV/AIDS, Viral Hepatitis Infectious Diseases 2013ZX10004-101 to CY) from Ministry of Science and Technology, People's Republic of China; and grant (2015SKLID507 to CY) from State Key Laboratory of Infectious Disease Prevention and Control, China CDC.

## Disclosure

YiWand CY have filed for a patent from the State Intellectual Property Office of the People's Republic China, which covers the novel assay and sequences included in this article (Application number CN201610576270.6).

### Conflict of interest statement

The authors declare that the research was conducted in the absence of any commercial or financial relationships that could be construed as a potential conflict of interest.
