# Supplementary figures and images for: Loop-Mediated Isothermal Amplification Label-Based Gold Nanoparticles Lateral Flow Biosensor for Detection of Enterococcus faecalis and Staphylococcus aureus
Source: Front Microbiol. 2017 Feb 10;8:192. doi: 10.3389/fmicb.2017.00192 (PMC5300967; doi:10.3389/fmicb.2017.00192)

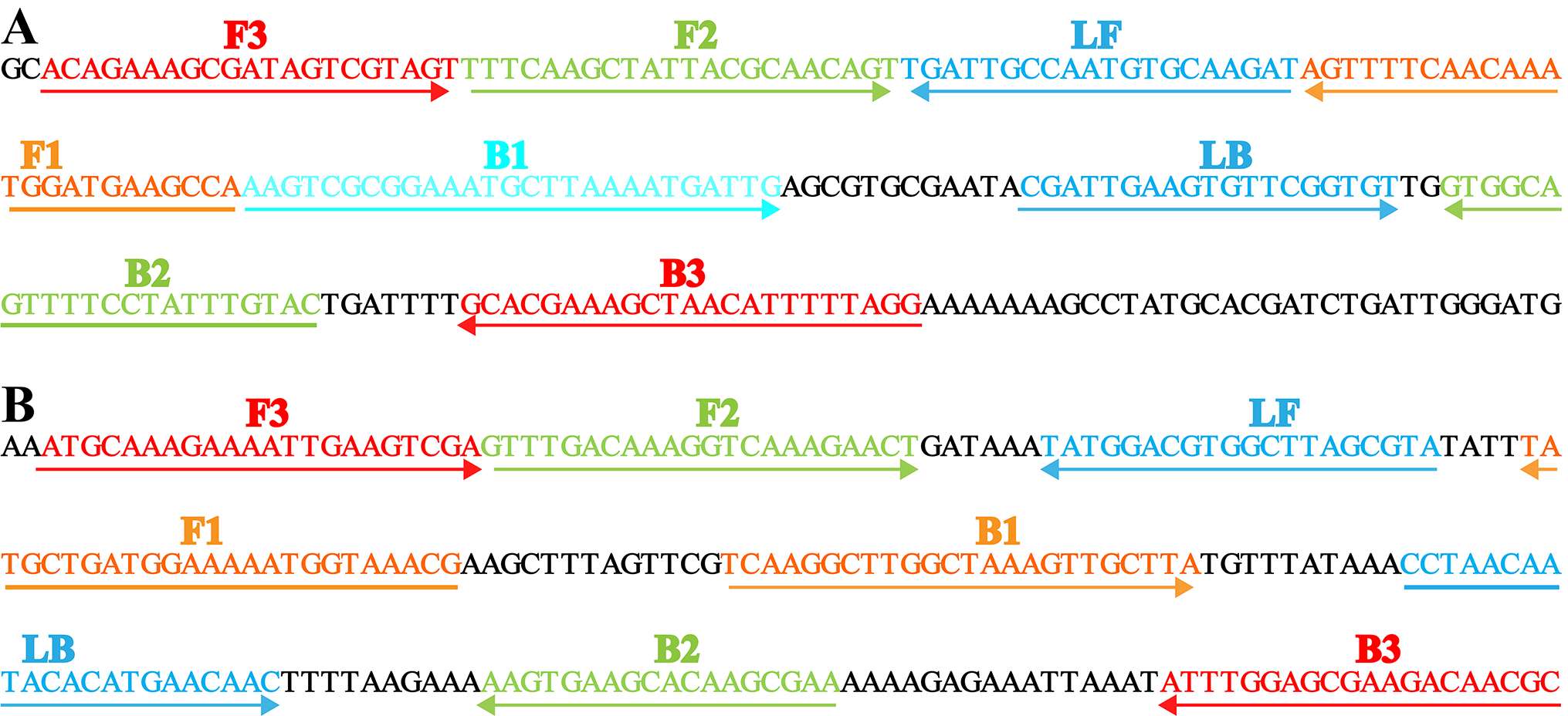

Supplement: Supplementary file 1 [file Image1.TIF]

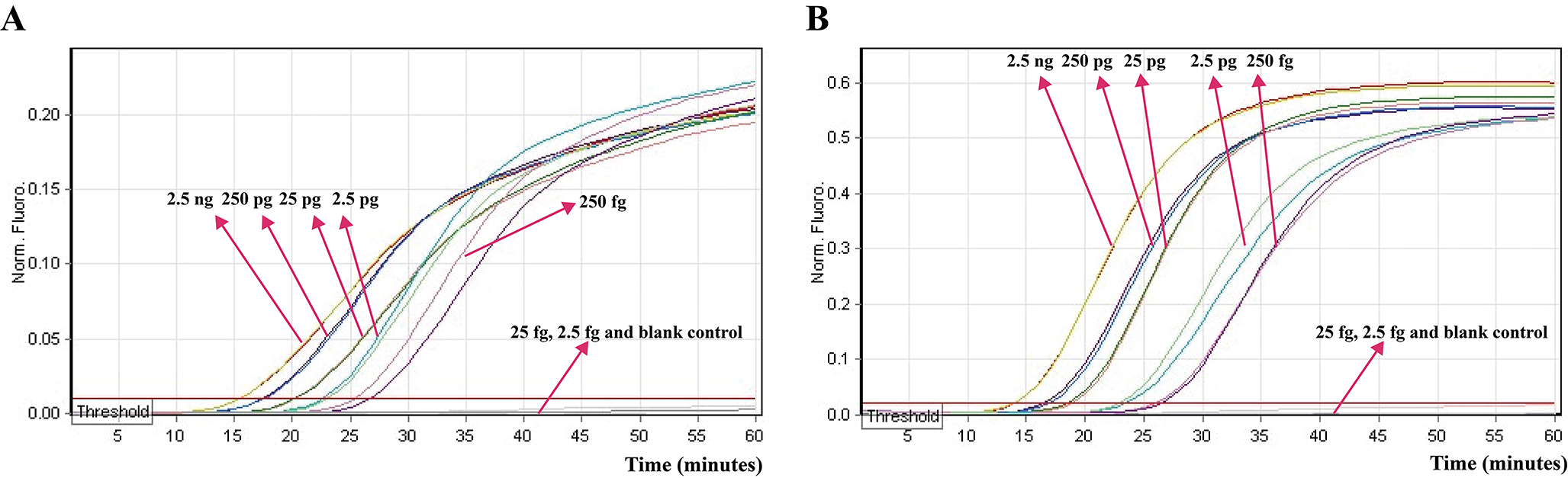

Supplement: Supplementary file 2 [file Image2.TIF]

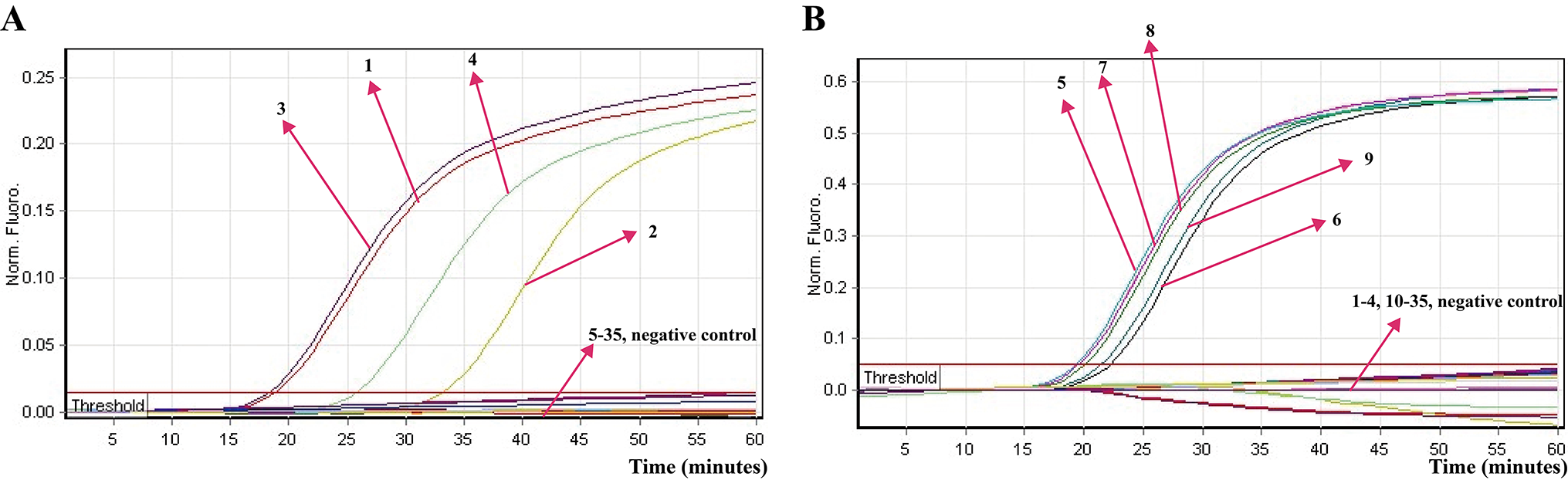

Supplement: Supplementary file 3 [file Image3.TIF]

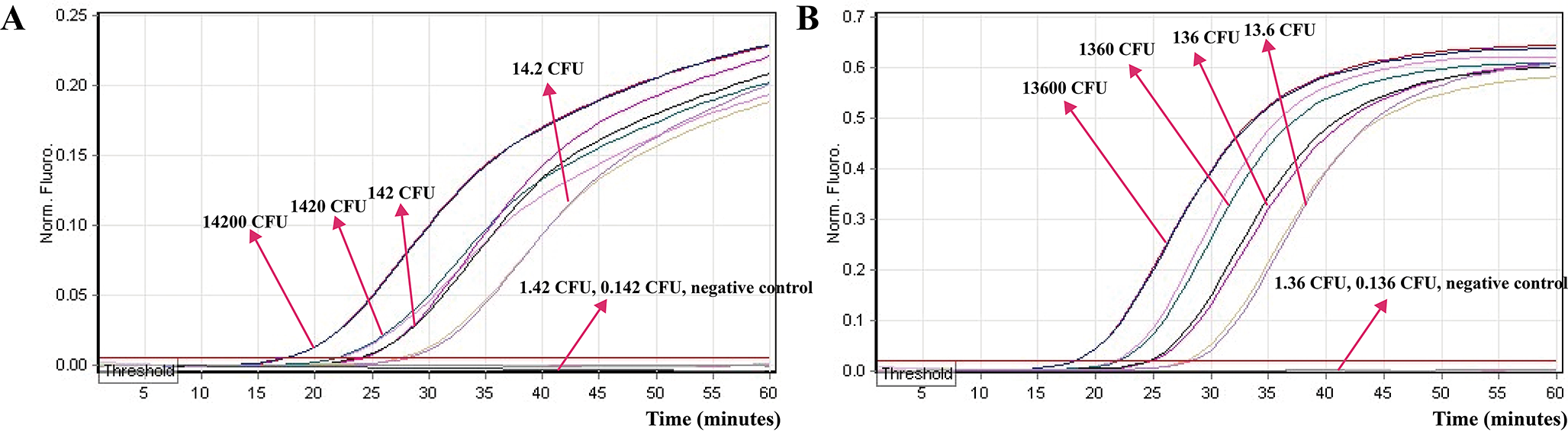

Supplement: Supplementary file 4 [file Image4.TIF]
