# Supplementary material for: Loop-Mediated Isothermal Amplification Label-Based Gold Nanoparticles Lateral Flow Biosensor for Detection of Enterococcus faecalis and Staphylococcus aureus
Source: Front Microbiol. 2017 Feb 10;8:192. doi: 10.3389/fmicb.2017.00192 (PMC5300967; doi:10.3389/fmicb.2017.00192)
Supplement: Supplementary file 5 [file DataSheet1.DOCX]

**Supplementary**

**Fig. S1 Location and sequences of *E. faecalis* (*Ef0027*) and *S. sureus* (*nuc*) genes used to design LAMP primers**

The nucleotide sequences of the sense strand of *Ef0027* (A) from *E. faecalis* and *nuc* (B) from *S. aureus* are shown. The DNA sequences used for primer targets are underlined. Right and left arrows indicate sense and complementary sequences that are used.

**Fig. S2** **Analysis of two targets in a multiplex MERT-LAMP reaction**

Two sets of MERT-LAMP primers targeting the *Ef0027* and *nuc* genes were simultaneously added into a single reaction, and the multiplex MERT-LAMP reactions were used for simultaneous detection of two targets: *E. faecalis* (A) and *S. aureus* (B). The 10-fold serial dilutions (2.5 ng, 250 pg, 25 pg, 2.5 pg, 250 fg, 25 fg and 2.5 fg/ul) of target templates were subjected to multiplex MERT-LAMP reactions, and two replicates of each dilution were tested to assess the analytical sensitivity of the MERT-LAMP amplifications. The corresponding curves of concentrations of DNA were marked in the figures. A and B were simultaneously generated from HEX (labeling EFIP of *Ef0027*) and Cy5 (labeling EFIP of *nuc*) channels. The fluorescence vs. time reaction curves was automatically yielded by instrument for *E. faecalis* (A) and *S. aureus* (B) detection.

**Fig. S3** The analytical specificity of multiplex MERT-LAMP detection of different strains

The multiplex MERT-LAMP reactions were conducted using different genomic templates and were analyzed by means of real-time detection. A and B were simultaneously obtained from HEX and Cy5 channels, respectively. Signals 1-3, strains of *E. faecalis* (ATCC 51299), *E. faecalis* (ICDC-NPEf001), *E. faecalis* (ICDC-NPEf002); signals 4-9, *S. aureas* (ICDC-NPSau001), *S. aureas* (ICDC-NPSau002), *S. aureas* (ICDC-NPSau003), *S. aureas* (ICDC-NPSau004), *S. aureas* (ICDC-NPSau005), *S. aureas* (Isolated strain); signals 13-35, non-*S. aureas*, non-*E. faecalis* strains of *Enterococcus faecium* (ATCC BAA340), *Staphylococcus epidermidis*, *Staphylococcus saparophytics*, *Enterococcus hirae, Streptococcus suis*, *Streptococcus pneumonia*, *Aeromonas hydrophila*, *Klebsiella pneumonia*, *Enteroinvasive E. coli*, *Enteropathogenic E. coli*, *Enterotoxigenic E. coli*, *Enteroaggregative E. coli*, *Enterohemorrhagic E. coli*, *Acinetobacter baumannii*, *Streptococcus sanguis*, *Streptococcus bovis*, *Pseudomonas aeruginosa*, *Campylobacter jejuni*, *Bntorobater sakazakii*, *Citro freumdii*, *Bacillus cereus*, *Shigella flexneri* and *Listeria monocytogenes*; signals 36, negative control.

**Fig. S4** **Analysis of two targets in spiked blood samples**

Two sets of MERT-LAMP primers targeting the *Ef0027* and *nuc* genes were simultaneously added into a single reaction, and the multiplex MERT-LAMP reactions were used for simultaneous detection of two targets in spiked blood samples: *E. faecalis* (A) and *S. aureus* (B). The 10-fold serial dilutions of target templates were subjected to multiplex MERT-LAMP reactions, and two replicates of each dilution were tested to assess the analytical sensitivity of the MERT-LAMP amplifications. The corresponding curves of concentrations of DNA were marked in the figures. A and B were simultaneously generated from HEX and Cy5 channels. The fluorescence vs. time reaction curves were automatically yielded by instrument for *E. faecalis* (A) and *S. aureus* (B) detection.
